# Planning Renal Replacement Therapy in Factor VII Deficiency

**DOI:** 10.1016/j.ekir.2025.11.023

**Published:** 2025-11-24

**Authors:** Ahmet Murt, Ertugrul Erol, Sefa Ergun, Muhlis Cem Ar

**Affiliations:** 1 Department of Nephrology, Cerrahpasa Medical Faculty, Istanbul University-Cerrahpasa, Istanbul, Turkey; 2 Department of General Surgery, Cerrahpasa Medical Faculty, Istanbul University-Cerrahpasa, Istanbul, Turkey; 3 Department of Hematology, Cerrahpasa Medical Faculty, Istanbul University-Cerrahpasa, Istanbul, Turkey

To the Editor:

With advances in long-term treatment strategies, patients with congenital coagulation factor deficiencies (i.e., hemophilia) are living longer but increasingly developing end-stage kidney disease.^1^^,^^2^ Selecting an optimal renal replacement therapy in this population remains clinically challenging.

Although successful renal transplantation has been reported in patients with hemophilia under adequate factor replacement therapy,^3^ surgical interventions continue to carry substantial bleeding risks. Hemodialysis may be feasible; however, repeated arterio-venous fistula cannulation often necessitates postdialysis factor infusions to achieve hemostasis, increasing both the cost and potential for inhibitor development.^4^^,^^5^ Peritoneal dialysis (PD) may be preferable, because bleeding episodes are uncommon and factor replacement is generally required only during catheter insertion.

We recently managed a 43-year-old female patient with congenital factor 7 deficiency who progressed to end-stage kidney disease. Her clinical course and the potential causes of chronic kidney disease in patients with coagulation disorders are provided in Supplementary Material S1 and S2.

Her sister was identified as a potential living kidney donor; however, routine pretransplant screening revealed the same factor 7 deficiency, thereby precluding transplantation. Currently, no guideline-based recommendations address dialysis modality selection in patients with coagulation factor deficiencies. Given this patient’s intermittent unexplained decreases in hemoglobin levels and increased risk of bleeding during hemodialysis, PD was considered a safer option. The patient was thoroughly counselled on PD, including relative merits of surgical versus percutaneous catheter insertion. A comparison of the advantages and disadvantages of percutaneous versus laparoscopic or surgical PD catheter insertion is provided in Table 1. She underwent percutaneous PD catheter placement performed by the nephrology team with periprocedural hemostatic support. The procedure was completed without complications, with only minor hemorrhagic fluid noted in the initial peritoneal effluent. Since then, the patient has been maintained on PD for a year without any significant complications. Strategies to plan renal replacement therapy in hemophiliacs is discussed in Supplementary Material S3.

**Table 1:** Pros and cons of percutaneous versus laparoscopic insertion of peritoneal dialysis catheter

Percutaneous insertion	Laparoscopic/surgical insertion
**Pros:**	**Pros:**
No need for general anesthesia	Direct observation of abdominal structures
No need for elective orotracheal intubation	Adhesiolysis may be performed
Less invasive	**Cons:**
Faster recovery	More invasive
**Cons:**	General anesthesia needed
No direct observation of abdominal structures	Orotracheal intubation needed
Patient compliance is needed	Prolonged recovery time

## Disclosure

AM declares receiving honorarium from Vantive, outside of this work. All the other authors declared no competing interests.

## Patient Consent

The authors declare that they have obtained consent from the patients discussed in the report. Patient identity is blinded and all rules of the Helsinki Declaration are obeyed.

## Data Availability Statement

All data of this report are available from the corresponding author upon a reasonable request.

## Supplementary Material

Supplementary File (PDF) **Supplementary References. S1.** Clinical history of the patient. **S2.** Reasons of chronic kidney disease in hemophiliacs. **S3.** Strategies to choose appropriate renal replacement therapy in hemophiliacs.

## Footnotes

Supplementary File (PDF)
Supplementary References. **S1.** Clinical history of the patient. **S2.** Reasons of chronic kidney disease in hemophiliacs. **S3.** Strategies to choose appropriate renal replacement therapy in hemophiliacs.
