# Supplementary material for: Planning Renal Replacement Therapy in Factor VII Deficiency
Source: Kidney Int Rep. 2025 Nov 24;11(2):103698. doi: 10.1016/j.ekir.2025.11.023 (PMC12769387; doi:10.1016/j.ekir.2025.11.023)
Supplement: Supplementary File (PDF) — Supplementary References. S1. Clinical history of the patient. S2. Reasons of chronic kidney disease in hemophiliacs. S3. Strategies to choose appropriate renal replacement therapy in hemophiliacs. [file mmc1.pdf]

Supplementary Material

S1. Clinical history of the patient

A 43 years old female patient had been under regular follow-up at the Hematology Outpatient Clinic of Cerrahpaşa Medical Faculty for several years. Her FVII activity level was measured at 0,4%, consistent with a severe deficiency. She received episodic replacement therapy with recombinant activated factor VII (rFVIIa) to treat her bleeding episodes. Approximately 18 months ago, she sustained a traumatic injury to her right shoulder, after which she required frequent rFVIIa replacement therapy and was prescribed non-steroidal anti-inflammatory drugs (NSAIDs) for pain management. Her creatinine level at that time was 0,8 mg/dL. Six months following the trauma, she presented to the emergency department with severe nausea and vomiting. Laboratory investigations revealed markedly elevated urea and creatinine levels—154 mg/dL and 4.42 mg/dL, respectively. A comprehensive diagnostic work-up did not identify any etiology for her acute kidney injury other than prolonged NSAID use. Due to concerns over post-procedural bleeding, a renal biopsy was not performed. Empirical corticosteroid therapy was initiated but yielded no clinical improvement. She was subsequently managed as a case of stage 5 chronic kidney disease (CKD), and within another six months, renal replacement therapy (RRT) became necessary. Despite receiving factor replacement therapy following her shoulder injury, the patient had experienced intermittent, unexplained drops in haemoglobin levels. A kidney transplantation was not possible for this patient because the patient's intended living donor—her sister—was also diagnosed with FVIID, raising significant concerns about perioperative bleeding risks for both donor and recipient. Among dialysis modalities, peritoneal dialysis (PD) was more convenient for this patient as haemodialysis may be related to more bleeding episodes. On the day of PD catheter placement, urea was 115 mg/dL, creatinine was 8,38 mg/dL and INR was 6,30. rFVIIa (30 mcg/kg) was administered prior to the procedure and INR dropped to 1,55. To enable better haemostasis, 15 mcg desmopressin was also given prior to the procedure.

S2. Reasons of chronic kidney disease in haemophiliacs.

CKD is among the comorbidities that may be observed during the follow-up of patients with congenital coagulation disorders Recurrent bleeding into urinary tract, microthrombi in the renal capillaries following factor replacements, repeated hypotensive events following major

bleeding, the use of nephrotoxic agents such as NSAIDs, and blood-borne viruses that may be transmitted via transfusions, such as hepatitis C and HIV, may contribute to the development and progression of CKD in patients with factor deficiencies.

S3: Strategies to choose appropriate renal replacement therapy in haemophiliacs

As the best RRT for the general CKD population, kidney transplantation remains a potential treatment option and successful outcomes under recombinant factor replacements, including FVII have been reported in the literature [S1, S2]. However, this was not rational in our case, as the patient's intended living donor—her sister—was also diagnosed with FVIID, raising significant concerns about perioperative bleeding risks for both donor and recipient. Given the hereditary nature of most coagulation factor deficiencies, potential living-related donors may themselves be affected, and this should be carefully investigated.

Currently, there are no guideline-based recommendations specifically addressing most appropriate dialysis modality in patients with coagulation factor deficiencies. Therefore, treatment decisions must be individualized, considering the patient's clinical characteristics and preferences. There have been previous reports of haemodialysis in haemophiliacs with AVFs, however patients had to receive factor infusions at the end of dialysis sessions to maintain haemostasis after removal of the needles. Such frequent factor infusions may be a reason of inhibitor development which might make bleeding control more difficult. Also, more frequent factor infusions may result in increased expenses. Central venous catheters may be used as vascular access but the risk of blood-borne infections limit their use. PD may offer a safer alternative to haemodialysis, as it is associated with reduced blood loss and may require less frequent factor replacement. Prior studies, albeit with small sample size, showed that PD was safe and effective in patients with haemophilia [S3]. However, patient compliance should be high, as PD is a modality that needs more active involvement of the patients.

Even when PD is chosen as an option with less bleeding, this choice does not fully eliminate procedural challenges. Catheter insertion itself poses a bleeding risk and must be approached with caution. In our case, we opted for percutaneous catheter placement under local anesthesia to avoid the additional risks associated with general anaesthesia and intubation. The percutaneous procedure was completed successfully without complications. Relevant

factor administrations and other preventive measures such as use of fresh frozen plasma or vasopressin generally decreases the risk of peri-procedural bleeding.

S4. Supplementary References

S1. Sauhta P, Gulati S, Narula AS, Gulia A, Shrikhande M, Gupta A. A Successful Kidney Transplant in a Patient With Hemophilia C. Exp Clin Transplant. 2024 Nov;22(11):883-885

S2. Abdeltawab K, Yagan J, Megahed M, et al. Kidney Transplant in a Patient With Factor VII Deficiency: Case Report. Exp Clin Transplant. 2019 Jan;17(Suppl 1):142-144.

S3. Zhang H, Wang H, Zhou Z, et al. End Stage Renal Failure Patients With Hemophilia Treated With Peritoneal Dialysis: A Case Series. Kidney Int Rep. 2022 Sep 30;7(12):2639-2646
